# Prevalence and correlates of fatigue and its association with quality of life among clinically stable older psychiatric patients during the COVID-19 outbreak: a cross-sectional study

**DOI:** 10.1186/s12992-020-00644-6

**Published:** 2020-12-18

**Authors:** Siyun Zou, Zi-Han Liu, Xiaona Yan, Huan Wang, Yulong Li, Xiuying Xu, Xiangdong Du, Lan Zhang, Qinge Zhang, Todd Jackson, Gabor S. Ungvari, Yu-Tao Xiang

**Affiliations:** 1 Medical College of Soochow University, Suzhou, Jiangsu Province China; 2 Guangji Hospital Affiliated to Soochow University, Suzhou, Jiangsu Province China; 3 Unit of Psychiatry, Institute of Translational Medicine, Faculty of Health Sciences, University of Macau, Building E12, Avenida da Universidade, Taipa, Macau SAR, China; 4 Center for Cognition and Brain Sciences, University of Macau, Macao SAR, China; 5 Institute of Advanced Studies in Humanities and Social Sciences, University of Macau, Macao SAR, China; 6 Department of Psychiatry, Xiamen Xianyue Hospital, Xiamen, China; 7 Department of Psychiatry, Lanzhou University Second Hospital, Lanzhou, Gansu Province China; 8 The National Clinical Research Center for Mental Disorders & Beijing Key Laboratory of Mental Disorders Beijing Anding Hospital & the Advanced Innovation Center for Human Brain Protection, Capital Medical University, School of Mental Health, Beijing, China; 9 Department of Psychology, University of Macau, Macau SAR, China; 10 Division of Psychiatry, School of Medicine, University of Western Australia, Perth, Australia; 11 University of Notre Dame Australia, Fremantle, Australia

**Keywords:** Fatigue, Quality of life, Older psychiatric patients, COVID-19

## Abstract

**Background:**

The pattern of fatigue in older psychiatric patients during the COVID-19 outbreak was unknown. This study examined the prevalence of fatigue and its association with overall quality of life (overall QOL) in clinically stable older patients with psychiatric disorders during the COVID-19 outbreak.

**Methods:**

This was a multicenter, cross-sectional study. Fatigue, depressive symptoms, pain, insomnia symptoms, and overall QOL were assessed with standardized instruments.

**Results:**

A total of 1063 patients were recruited. The prevalence of fatigue was 47.1% (95%CI: 44.1–50.1%). An analysis of covariance revealed that overall QOL was significantly lower in patients with fatigue compared to those without (*P* = 0.011). A multiple logistic regression analysis revealed that more severe depressive symptoms (OR = 1.15, *P* < 0.001), insomnia symptoms (OR = 1.08, *P* < 0.001) and pain (OR = 1.43, *P* < 0.001) were significantly associated with fatigue.

**Conclusions:**

Fatigue is common among clinically stable older patients with psychiatric disorders during the COVID-19 outbreak. Considering its negative impact on overall QOL, regular assessment of fatigue and appropriate treatment warrant attention in this subpopulation.

## Background

Since the novel coronavirus disease 2019 (COVID-19) was first identified in Wuhan, China in December, 2019, it has been found in more than 200 countries and territories globally [55]. Mental health symptoms and related problems in different subpopulations have emerged as negative health outcomes caused by the COVID-19 pandemic [48]. Compared to the general population, patients with psychiatric disorders are more likely to experience psychological distress during the COVID-19 pandemic. Psychiatric patients could also have a higher risk of contagion due to limited awareness of self-protection, and non-adherence to preventive public health measures [65]. Similar to patients with chronic physical illnesses, clinically stable psychiatric patients require long-term maintenance pharmacotherapy and must, therefore, regularly travel from their residences to outpatient clinics [58, 60]. However, social distancing, transportation restrictions and potential infection risks during the COVID-19 pandemic increase inconvenience and potentially compromise safety for those who have to attend psychiatric outpatient appointments [31].

Compared to their younger counterparts, older psychiatric patients are at a higher risk of COVID-19 [4] due to severe cognitive decline, poor adherence to preventive measures, poor general health, and common severe physical diseases [62]. During the COVID-19 pandemic, public transportation in many areas has been suspended and online mental health services have been widely adopted [32]. However, due to limited access to internet services and smart phones, only a small fraction of older adults can benefit from such online service provision [12]. All of these factors could trigger pre-existing psychiatric disorders and increase risk for physical and psychological distress in older psychiatric patients.

Fatigue symptoms (Fatigue hereafter) are characterized by persistent weakness or exhaustion that usually occurs in tandem with impaired cognition, physical pain and sleep problems [24]. Fatigue is a common complaint in older adults [40]. Apart from the general population, patients with psychiatric disorders such as depression, anxiety, panic, eating disorders, substance misuse disorders, and somatisation disorders often suffer from fatigue [47]. For example, the prevalence of fatigue in patients with depression or anxiety ranges from 25 to 36% [20, 37, 52]. Fatigue is associated with a range of negative health outcomes including sleep disturbances [27] and poor general health status [15]. Recent studies have found that fatigue has been common in many subgroups during the COVID-19 pandemic such as patients confirmed with COVID-19 (53.6%) [44], medical workers [46], and students [35]. Such effects are partially due to lockdown, quarantine, social distancing, and unprecedented pressure in daily life. Previous studies mainly focused on the mental impactof the COVID-19, such as low wellbeing, neuropsychiatric symptoms, and declinedcognition in older psychiatric patients [11, 21, 36], but fatigue in thispopulation was not reported worldwide.. Furthermore, to date, the impact of fatigue on quality of life (QOL), a widely used comprehensive health outcome, of older psychiatric patients is unknown. Similarly, links between fatigue and other experiences common to this population (e.g., depressive symptoms, insomnia symptoms, and pain) warrant consideration.

Therefore, we conducted this study to investigate the prevalence of fatigue and its associations with QOL as well as depressive symptoms, insomnia symptoms, and pain in clinically stable older patients with psychiatric disorders during the COVID-19 pandemic. We hypothesized that the prevalence of fatigue in older psychiatric patients during the COVID-19 pandemic would be higher than that in general older adult population in China. In addition, depressive symptoms, insomnia symptoms and pain were expected to be associated with fatigue in older psychiatric patients during COVID-19 pandemic. Third, patients with fatigue would have a lower overall QOL than those without fatigue.

## Methods

### Setting and sample

This cross-sectional study was carried out between May 22 and July 15, 2020 in outpatient geriatric psychiatry clinics of four major tertiary psychiatric hospitals/centres in China (i.e., Beijing Anding Hospital, Lanzhou University Second Hospital, Guangji Hospital Affiliated to Soochow University, and Xiamen Xianyue Hospital). Patients who received maintenance treatment in the participating hospitals were consecutively enrolled. The inclusion criteria were as follows: 1) aged 50 years and older [59]; 2) diagnosed with psychiatric disorders according to the International Statistical Classification of Diseases and Related Health Problems, 10th Revision (ICD-10) [54]; 3) clinically stable based on the judgment of treating psychiatrists; and 4) able to understand and read Chinese and provide written informed consent.

All participants who agreed to participate provided written informed consent forms. This study was approved by the ethical committees of respective hospitals. All study procedures were carried out in accordance with relevant ethical guidelines in participating hospitals.

### Instruments

Basic demographic and clinical characteristics including age, gender, residence, marital status, educational level, severe physical illnesses, fluctuations of psychiatric disorders, frequent use of mass media, limited access to psychiatric services, poor treatment adherence during the COVID-19 pandemic, and concern about COVID-19 (i.e., whether or not the patient was concerned of the COVID-19 related information during the pandemic) were collected. Due to risk of transmission during the COVID-19 outbreak, face-to-face interviews were not performed. Following other studies [34], the WeChat-based QuestionnaireStar program, a commonly used application for epidemiological surveys [28, 56], was used to collect data. WeChat is a widely used social communication program with more than 1 billion users in China including older patients enrolled in this research and/or their guardians.

Severity of current fatigue was assessed using a numeric rating scale (NRS) [3] from “0” (no suffering from fatigue) to “10” (unbearable suffering from fatigue) [2]. Total scores of ≥4 were considered “having clinically relevant fatigue” (“having fatigue” hereafter) [43]. The self-report 9-item Patient Health Questionnaire (PHQ-9), Chinese version, was used to measure severity of depressive symptoms [6, 26, 61]. Each item was scored from “0” (not at all) to “3” (nearly every day). The Chinese version of PHQ-9 had satisfactory psychometric properties in older Chinese samples with the Cronbach’s alpha of 0.83 [61]. Severity of insomnia symptoms was assessed with the 7-item Insomnia Severity Index (ISI) [41], with each item scoring from “0” (not at all) to “4” (severe) [49]. The ISI has satisfactory psychometric properties in Chinese older population with the Cronbach’s alpha of 0.81 [64]. The numeric Pain Rating Scale (NPRS) was used to assess severity of pain [18]; “0” (no pain) and “10” (worst pain imaginable) were used as anchors. The Chinese version of NPRS has been widely used in different populations [29, 30, 33]. The sum of the first two items of the World Health Organization Quality of Life-brief version (WHOQOL-BREF) [13, 19, 57] was calculated to assess patients’ overall QOL, with higher scores indicating a higher overall QOL. Data on frequent use of mass media, limited access to psychiatric services, fluctuations of psychiatric disorders, concern about COVID-19 and poor treatment adherence during COVID-19 outbreak were collected from patient self-reports or their guardian’s reports; fluctuations of psychiatric disorders and poor treatment adherence were confirmed by treating psychiatrists.

### Data analysis

Data were analysed using the IBM Statistical Package for Social Science (SPSS) program, version 23.0. A P-P Plot analysis was performed to test normality of continuous variable distributions. To compare demographic and clinical characteristics between fatigue and no fatigue groups, Chi-square tests, independent samples t tests and Mann-Whitney U Tests were used, as appropriate. A multiple logistic regression analysis, with the “Enter” method, was conducted to examine independent demographic and clinical correlates of fatigue; fatigue was the dependent variable and other measures that had *P* values of < 0.05 in univariate analyses were independent variables. To compare overall QOL differences between fatigue and no fatigue groups, an analysis of covariance (ANCOVA) was conducted after controlling for variables that had statistically significant differences in univariate analyses. The significance level was set at *P* < 0.05 (two-tailed).

## Results

In total, 1068 patients were invited, of whom, 1063 met the study entry criteria and joined this study. The prevalence of fatigue (total score of ≥4) was 47.1% (95%CI: 44.1–50.1%) with a mean fatigue total score of 3.4 (SD = 2.7). Table 1 shows demographic and clinical characteristics of participants.

**Table 1:** Socio-demographical and clinical characteristics of the participants

**Variable**	**Total**		**Fatigue**		**No Fatigue**		**Univariate analyses**		
	**(*N*** **= 1063)**		**(*N*** **= 501)**		**(*N*** **= 562)**				
	**N**	**%**	**N**	**%**	**N**	**%**	***χ***^**2**^	**df**	***P***
Male gender	347	32.6	157	31.3	190	33.8	0.74	1	0.391
Married	961	90.4	446	89.0	515	91.6	2.09	1	0.148
Rural residence	373	35.1	203	40.5	170	30.2	12.27	1	**< 0.001**
Have severe physical illness	190	17.9	104	20.8	86	15.3	5.37	1	**0.020**
Concerned about COVID-19	744	70.0	347	69.3	397	70.6	0.24	1	0.624
Frequent use of mass media during COVID-19 outbreak	215	20.2	99	19.8	116	20.6	0.13	1	0.721
Limited access to psychiatric services during COVID-19 outbreak	367	34.5	193	38.5	174	31.0	6.70	1	**0.010**
Poor treatment adherence during COVID-19 outbreak	365	34.3	187	37.3	178	31.7	3.75	1	0.053
Illness worsening during COVID-19 outbreak	564	53.1	345	68.9	219	39.0	95.04	1	**< 0.001**
Principal psychiatric diagnosis							13.17	1	**< 0.001**
MDD	485	45.6	258	51.5	227	40.4			
Other diagnoses ^b^	578	54.4	143	48.5	335	59.6			
**Variable**	**Mean**	**SD**	**Mean**	**SD**	**Mean**	**SD**	***t/Z***	**df**	***P***
Age (years)	62.80	9.44	62.41	9.48	63.15	9.40	−1.28	1061	0.202
Education (years)	7.96	4.03	7.51	4.04	8.37	3.98	−3.94	-^a^	**< 0.001**
PHQ-9 total score	7.76	6.73	11.40	6.78	4.51	4.73	−17.28	- ^a^	**< 0.001**
ISI total score	8.93	6.36	11.84	6.11	6.34	5.40	−14.26	- ^a^	**< 0.001**
Pain total score	1.53	2.20	2.33	2.59	0.82	1.47	−9.78	- ^a^	**< 0.001**
Overall quality of life	6.26	1.56	5.69	1.55	6.77	1.38	−12.08	1061	**< 0.001**

Bolded values: < 0.05; *COVID-19* Coronavirus Disease 2019, *MDD* Major depressive disorder, *PHQ-9* the 9-item Patient Health Questionnaire, *ISI* Insomnia Severity Index, *SD* Standard deviation. ^a^ Mann-Whitney U Test; ^b^ other diagnoses included bipolar disorder, schizophrenia, organic mental disorders, etc. P-P plots revealed that among the six continuous variables (e.g., age, education years, PHQ-9 total score, ISI total score, pain total score, and overall QOL), age and overall QOL scores were normally distributed but other continuous variables had skewed distributions

Univariate analyses revealed that fatigue was significantly associated with rural residence (*P* < 0.001), having severe physical illness (*P* = 0.02), limited access to psychiatric services (*P* = 0.01), illness worsening during COVID-19 outbreak (*P* < 0.001), having the diagnosis of major depressive disorder (*P* < 0.001), fewer years of education (P < 0.001), and higher total scores on the PHQ-9 (*P* < 0.001), ISI (*P* < 0.001), and NPRS (*P* < 0.001). After controlling for covariates, clinically stable older psychiatric patients with fatigue reported lower overall QOL compared than those without fatigue (F _(1, 1063)_ = 6.471, *P* = 0.011).

Multiple logistic regression analysis revealed more severe depressive symptoms (OR = 1.15, *P* < 0.001), insomnia symptoms (OR = 1.08, *P* < 0.001) and pain (OR = 1.43, *P* < 0.001) were significantly associated with fatigue (Table 2).

**Table 2:** Independent correlates of fatigue by multiple logistic regression analysis

Variables	Multiple logistic regression analysis		
	***P*** value	OR	95% CI
Rural residence	0.66	1.09	0.75–1.57
Presence of severe physical illness	0.628	1.11	0.72–1.71
Limited access to psychiatric services during COVID-19 outbreak	0.549	1.11	0.79–1.58
Illness worsening during COVID-19 outbreak	0.237	1.22	0.88–1.70
Principal psychiatric diagnosis (MDD)	0.075	1.34	0.97–1.84
Education (years)	0.072	0.96	0.92–1.00
PHQ-9 total score	**< 0.001**	1.15	1.11–1.19
ISI total score	**< 0.001**	1.08	1.05–1.12
Pain total score	**< 0.001**	1.43	1.30–1.57

Bolded values: < 0.05; *CI* Confidential interval, *OR* Odds ratio, *MDD* Major depressive disorder, *PHQ-9* the 9-item Patient Health Questionnaire, *ISI* Insomnia Severity Index. Participating hospitals were controlled for as covariate

## Discussion

This was the first study that examined the prevalence of fatigue in older psychiatric patients and its impact on overall QOL during the COVID-19 outbreak. We found that 47.1% (95%CI: 44.1–50.1%) of clinically stable older patients with psychiatric disorders reported fatigue during the COVID-19 outbreak, which is higher than the prevalence (31.2%, 95%CI: 30.0–32.5%) observed using standardized questions in the general older adults in China [40], and similar to the rate (53.6%) found using the Fatigue Scale-14 in COVID-19 patients [44]. Although potentially informative, direct comparisons between studies using different measures of fatigue should be made with caution.

The high prevalence of fatigue in clinically stable older psychiatric patients could be due to several reasons. First, fatigue is a comorbid symptom of many psychiatric diagnoses. For instance, a study found that 60% of psychiatric inpatients reported significant level of fatigue [53], particularly those with depressive or anxiety disorders [50]. Although patients were clinically stable during the study period, the COVID-19 outbreak and related problems such as limited access to psychiatric services and fear of transmission could have triggered certain comorbid symptoms such as fatigue, in these patients. Second, many older adults suffered from severe and/or chronic physical illnesses. Due to the suspension of public transportation and mass quarantine measures, this group may have had more difficulty attending follow-up appointments and receiving maintenance treatments, in turn, exacerbating their physical illnesses (e.g., cancer, hypertension, chronic pain disorder, chronic obstructive pulmonary disease) and experience of fatigue [50, 51]. Third, sedentary lifestyle, due to lack of physical exercise, was associated with fatigue [38]. As such, lack of outdoor activities and physical exercise due to quarantine measures during the COVID-19 pandemic could cause fatigue. Finally, the high mortality rate and poor prognosis of COVID-19 in older adults could lead to perceptions of health risks and psychological distress, which may have resulted in increased loneliness and fatigue [17]. Previous studies found that fatigue was positively associated with female gender and younger age [1, 14, 23], but these results were based on general samples and assessed under “non-pandemic” conditions. In this study, however, no significant associations between fatigue and gender or age were found.

As expected, patients with more severe depressive symptoms (OR = 1.15, *P* < 0.001), insomnia symptoms (OR = 1.08, P < 0.001) and more severe pain (OR = 1.43, *P* < 0.001) were more likely to report fatigue. Fatigue is common in depressed patients, particularly in those with comorbid somatic symptoms. Previous studies have found that many depressed patients complained of fatigue [8, 16, 39], an effect that may be partly due to common underlying genetic factors [10]. In addition, fatigue could be induced by antidepressant medications [38]. Some studies have found insomnia is a contributing factor to fatigue [27] and improvements in sleep problems as a correlate of decreases in fatigue complaints among older adults [9]. In this study, patients reporting more severe pain were at higher risk for fatigue, a finding that is consistent with previous findings in patients with primary Sjögren’s syndrome [7], cancer [45], or multiple sclerosis [25].

We found that patients with fatigue had a lower overall QOL than those without, consistent with previous findings [25, 42]. QOL is determined by interactions between protective factors (e.g., higher economic status) and risk factors (e.g., poor physical health) [22]. Fatigue is associated with sleep disturbances, reduced energy and motivation, and cognitive dysfunctions, all of which could negatively affect daily functioning, increase physical and mental distress, and eventually lower QOL [5, 15, 63].

The strengths of this study included its focus on an understudied, at-risk population, multi-center design, use of validated measures, and large sample size. However, several methodological limitations should also be acknowledged. First, because we focused on clinically stable older patients, findings cannot be generalized to patients in other illness stages. Second, causal relations between fatigue and other variables could not be established due to the cross-sectional study design. Third, for logistical reasons (i.e., concerns about high response burdens in a vulnerable group), some factors associated with fatigue (e.g., treatment of physical illnesses and economic status), were not examined in this study.

## Conclusion

In conclusion, this was the first study that examined the prevalence of fatigue in older psychiatric patients and its impact on overall QOL during the COVID-19 outbreak. The prevalence of fatigue was 47.1%, indicating that it is common among clinically stable older patients with psychiatric disorders during the COVID-19 pandemic. Given the prevalence of fatigue and its negative associations with overall QOL andother disturbances, regular screening onfatigue should be conducted in older psychiatric patients in all countriesaffected by the COVID-19 pandemic.Furthermore, routine assessment of the presence and severity of fatigue shouldbe conducted in this vulnerable population, and appropriate treatments shouldbe offered to those in need.

## Data Availability

The datasets used and/or analyzed during the current study are available from the corresponding author on reasonable request.

## Abbreviations

COVID-19: Novel coronavirus disease 2019
Overall QOL: Overall quality of life
PHQ-9: The self-report 9-item Patient Health Questionnaire
ISI: The 7-item Insomnia Severity Index
NPRS: Numeric pain rating scale
FS-14: Fatigue Scale-14
NRS: Numeric rating scale
WHOQOL-BREF: World Health Organization Quality of Life-brief version
SPSS: IBM Statistical Package for Social Science
ANCOVA: Analysis of covariance
